# A Quantitative Meta-Analysis and Qualitative Meta-Synthesis of Aged Care Residents’ Experiences of Autonomy, Being Controlled, and Optimal Functioning

**DOI:** 10.1093/geront/gnad135

**Published:** 2023-10-05

**Authors:** Emma L Bradshaw, Joel R Anderson, Ma A J Banday, Geetanjali Basarkod, Rafaan Daliri-Ngametua, Kelly A Ferber, Dylan Henry, Richard M Ryan

**Affiliations:** Faculty of Health Sciences, Institute for Positive Psychology and Education, Australian Catholic University, North Sydney, New South Wales, Australia; Faculty of Health Sciences, Institute for Positive Psychology and Education, Australian Catholic University, North Sydney, New South Wales, Australia; Australian Research Centre in Sex, Health and Society, La Trobe University, Bundoora, Victoria, Australia; Faculty of Health Sciences, School of Psychology, Australian Catholic University, Strathfield, New South Wales, Australia; Faculty of Health Sciences, Institute for Positive Psychology and Education, Australian Catholic University, North Sydney, New South Wales, Australia; Faculty of Education and Arts, School of Education, Australian Catholic University, Banyo, Queensland, Australia; Faculty of Health Sciences, Institute for Positive Psychology and Education, Australian Catholic University, North Sydney, New South Wales, Australia; Faculty of Health Sciences, School of Psychology, Australian Catholic University, Fitzroy, Victoria, Australia; Faculty of Health Sciences, Institute for Positive Psychology and Education, Australian Catholic University, North Sydney, New South Wales, Australia; Ewha Womans University, Seoul, South Korea

**Keywords:** Self-determination theory, Systematic review, Motivation, Nursing home

## Abstract

**Background and Objectives:**

The poor mental health of adults living in aged care needs addressing. Improvements to nutrition and exercise are important, but mental health requires a psychological approach. *Self-determination theory* finds that autonomy is essential to wellbeing while experiences of being controlled undermine it. A review of existing quantitative data could underscore the importance of autonomy in aged care, and a review of the qualitative literature could inform ways to promote autonomy and avoid control. Testing these possibilities was the objective of this research.

**Research Design and Methods:**

We conducted a mixed-methods systematic review of studies investigating autonomy, control, and indices of optimal functioning in aged care settings. The search identified 30 eligible reports (19 quantitative, 11 qualitative), including 141 quantitative effect sizes, 84 qualitative data items, and *N* = 2,668. Quantitative effects were pooled using three-level meta-analytic structural equation models, and the qualitative data were meta-synthesized using a grounded theory approach.

**Results:**

As predicted, the meta-analysis showed a positive effect of aged care residents’ autonomy and their wellness, *r* = 0.33 [95% CI: 0.27, 0.39], and a negative effect of control, *r* = −0.16 [95% CI: −0.27, −0.06]. The meta-synthesis revealed seven primary and three sub-themes describing the nuanced ways autonomy, control, and help seeking are manifest in residential aged care settings.

**Discussion and Implications:**

The results suggest that autonomy should be supported, and unnecessary external control should be minimized in residential aged care, and we discuss ways the sector could strive for both aims.

## Background and Objectives

Residential aged care—synonymous with nursing home care—refers to facilities that offer live-in, long-term care for older adults (Chow & Camões-Costa, 2021). Much of the Western world relies considerably on residential aged care (Dyer et al., 2020). Unfortunately, some adults in residential aged care have poor mental health (Cleland et al., 2021), especially compared to older adults living in community. Roughly 10%–15% of older adults experience depression, but for those living in aged care the rate is much larger (Blackburn et al., 2017). In some jurisdictions, aged care residents’ have been shown to have depression rates four times higher than community-dwelling older adults (Jongenelis et al., 2004). Clearly, concerted effort should be made to improve the wellbeing of adults living in aged care.

In addition to poor mental health due to ill health and social isolation (Davison et al., 2012), aged care residents often report experiencing “suboptimal care” and perceive few efforts to improve their circumstances (Walker & Paliadelis, 2016, p. E9). Despite these unfavorable perceptions, efforts *have* been made to reform the residential aged care sector (Zimmerman et al., 2014). For example, strides have been made in improving residents’ nutrition (Gaskill et al., 2008) and levels of physical activity (Sherrington et al., 2020). Although practical enhancements to things like food and exercise are essential to improving the sector, they may be insufficient to adequately support residents’ physical *and* mental health. Given the importance of residents’ physical *and* psychological wellbeing, psychological approaches should complement existing practical reforms.

Evidence from *self-determination theory* (SDT; Ryan & Deci, 2017) suggests how wellbeing in residential aged care could be improved. Within SDT, wellbeing is defined as optimal functioning and experience reflected in feelings of positivity, meaning, and satisfaction (Ryan & Deci, 2001). Wellbeing encompasses mental health indicators like vitality and lack of depression, as well as feeling physically well and purposeful. Self-determination theory research consistently demonstrates that people’s wellbeing depends fundamentally on their experiences of autonomy (heretofore, simply “autonomy”) and is undermined when people feel they are being controlled (heretofore, simply “control”; e.g., Ng et al., 2012). These experiences may be especially relevant to the physical and psychological health of adults in aged care (e.g., Altintas et al., 2017; Kloos et al., 2019), because their autonomy could be considered perpetually undermined.

Autonomy involves feeling volitional, agentic, and able to reasonably choose whether to enact behaviors (Weinstein et al., 2012). Experiences of autonomy are reflected in: (a) feeling autonomy supported—which means feeling heard, respected, and empowered (Reeve & Jang, 2006), (b) autonomous forms of motivation like intrinsic motivation—which reflects inherent enjoyment, and identified motivation—which reflects genuine valuing, and (c) in the satisfaction of humans’ basic psychological needs for autonomy, competence (i.e., capability and effectiveness), and relatedness (i.e., closeness with others; Donald et al., 2021). These experiences of autonomy have all been shown to bolster the physical and psychological health of aged care residents (Altintas et al., 2017; Davison et al., 2022; Vallerand et al., 1989).

Conversely, experiences of being controlled restrict autonomy not only via coercion and punishment, but also when voices and choices are ignored, which undermines the wellbeing of adults in aged care (Altintas et al., 2017). Control is represented by experiences like: (a) frustration of basic psychological needs, (b) amotivation (i.e., the absence of drive), and (c) controlled forms of motivation like external motivation—which reflects reliance external contingencies, and introjected motivation—which reflects internal pressure (Donald et al., 2021). Some control may be appropriate in aged care settings due to residents’ limited cognitive or physical capacities. Yet, *how* restrictions are imposed and thereby experienced by residents, can diminish autonomy, and increase feelings of being controlled, at a cost to individuals’ wellbeing.

Experiences of autonomy and control may also be relevant to residents’ abilities to rely on aged care staff. Adults’ entry into aged care is often necessitated by physical and cognitive limitations (Gibson, 2020). Thus, being able to rely on others is an essential part of healthy and effective adaptation to nursing home life. However, one’s tendency to willingly receive support—to be *autonomously reliant*—depends on the degree of autonomy support experienced within relationships (Deci et al., 2006). For example, children rely more autonomously on their parent/s if they perceive them as being autonomy supportive, with similar patterns demonstrated in romantic couples and best friendships (Ryan et al., 2005). The resident-carer dyad may not be marked by the same emotional closeness as a romantic or familial bond. Nonetheless, the evidence suggests that experiences of autonomy in aged care are strongly, positively linked with residents’ autonomous reliance (Altintas et al., 2017).

The literature on the effects of autonomy and control on aged care residents’ wellbeing—in psychological (e.g., life satisfaction), physical (e.g., general health), and contextual (e.g., autonomous reliance) domains—is now sufficiently developed to warrant a meta-analytic review of their direction, magnitude, and generalizability. This is the first aim of the present research. In a mixed-methods approach to our research aims, we also conducted a qualitative meta-synthesis of residents’ descriptions of autonomy, control, and autonomous reliance because the context-specific descriptions of these experiences are arguably still unclear. Meta-synthesis is a qualitative research method that involves integrating qualitative data from multiple primary studies to deliver higher-order, summative conclusions (Walsh & Downe, 2005). From cross-culturally validated measures of autonomy satisfaction, we know that “a sense of choice and freedom” and “doing what really interests me” (Chen et al., 2015, p. 227), effectively index people’s perceived autonomy. But how are these experiences best supported for people whose freedoms and choices are fundamentally limited and are subject to paternalism? In what specific ways are autonomy, control, and autonomous reliance manifest aged care? We argue that residents are the optimal source of such information, and thus, a qualitative meta-synthesis may be the key to leveraging the meta-analytic results into practical, tangible applications.

## The Present Study

**Figure 1.**
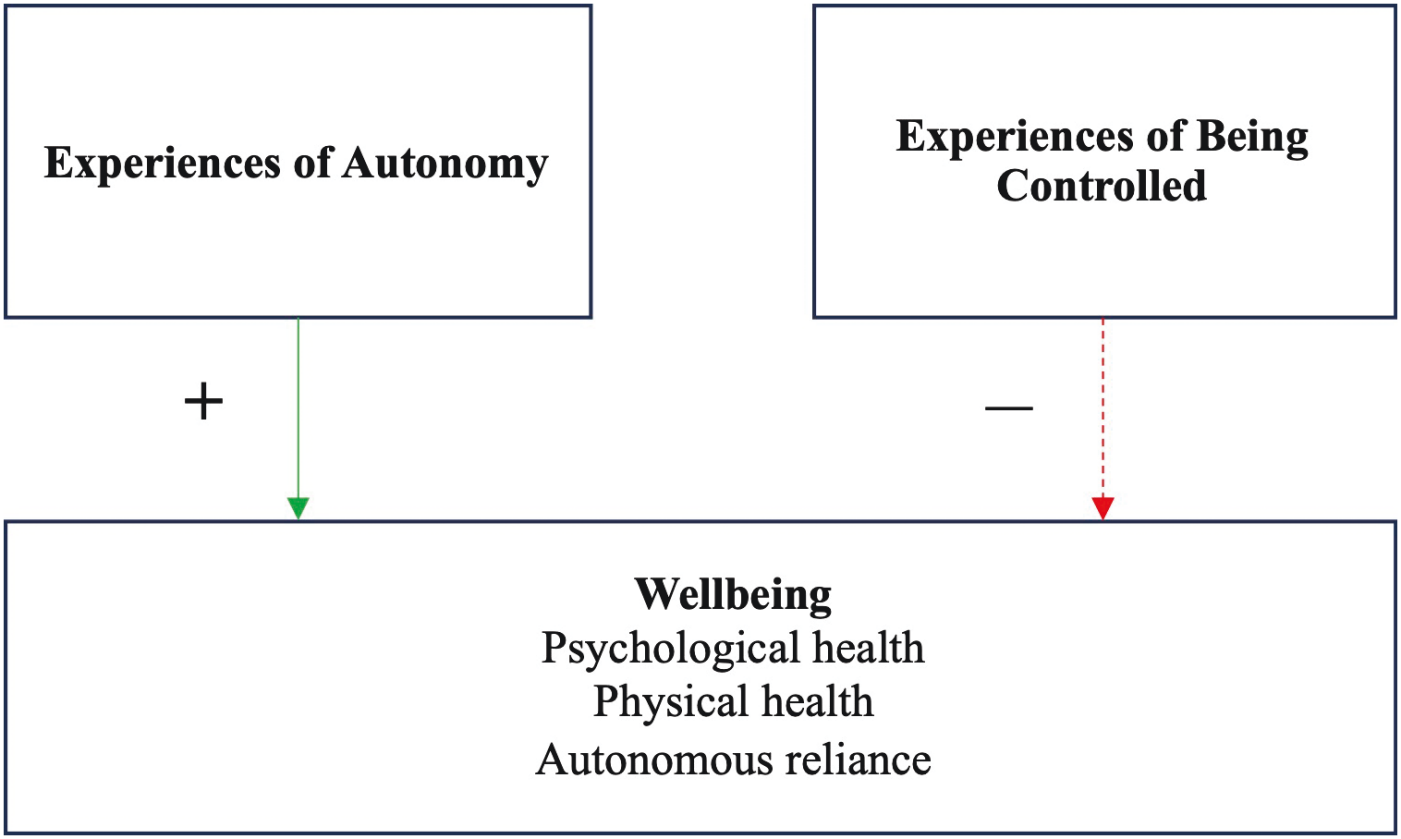
The self-determination theory-based model in which aged care residents’ experiences of autonomy are expected to positively link with their wellbeing and experiences of being controlled negatively link to wellbeing. The green, solid line indicates a hypothesized positive link (+), the dotted, red line signifies a hypothesized negative link (—).

Using a mixed-methods approach, we first conducted a meta-analysis of relevant quantitative data to examine if autonomy is positively linked (Hypothesis 1) and control is negatively linked (Hypothesis 2) with aged care residents’ wellbeing and autonomous reliance. Figure 1 illustrates these theoretically anchored main effects hypotheses. The meta-analysis will also test possible moderators including (a) the type of outcome (i.e., psychological wellbeing, physical wellbeing, and autonomous reliance), to see if autonomy and control are relevant to a specific outcome type, though we expect the effects to be relatively consistent across outcome types (Hypothesis 3). Then, (b) the specific experience of autonomy (i.e., autonomy support, autonomous motivations, and basic psychological need satisfaction) and specific experience of control (i.e., basic psychological need frustration, amotivation, and controlled motivations). Different experiences of autonomy should link relatively consistently with wellbeing outcomes (Hypothesis 4). However, controlled motivations such as amotivation and external motivation have been shown to be more detrimental than, for example, introjected motivation (Donald et al., 2020; Vasconcellos et al., 2020), so the type of control is likely to moderate the main effect of control on residents’ optimal functioning (Hypothesis 5).

We will also test demographic moderators including country, age, and proportion of females, though we expect these effects to be relatively stable or unmoderated (Hypothesis 6), consistent with SDT’s claims about the universal importance of autonomy and costs of control (see Bradshaw et al., 2022, 2023). In the second part of the study, we conducted a meta-synthesis of relevant qualitative data, using a grounded theory method (detailed in the Methods section below) to give voice to aged care residents, by deriving themes from previous qualitative research that convey specific examples of residents’ experiences of autonomy, control, and autonomous reliance.

## Research Design and Methods

### Registration and Open Science Practices

The study design for this systematic review was registered on the Open Science Framework on May 23, 2022 (https://doi.org/10.17605/OSF.io/vxkyc). The R code and data underlying the meta-analyses, and the extracted qualitative data, have also been made publicly available via the above link.

### Eligibility Criteria

To be included in the meta-analysis or meta-synthesis, studies needed to meet the below criteria: (a) a sample of older adults currently living in residential aged care; (b) the study used a valid quantitative measure of autonomy and/or control, or qualitatively assessed definitions of self-determination/autonomy, control, and/or autonomous reliance; and (c) the study used a valid quantitative measure of physical wellbeing, psychological wellbeing (or ill-being), and/or autonomous reliance.

For quantitative studies, the link between (b) and (c) needed to be reported or sourceable. Qualitative studies (and qualitative data from mixed-methods studies) did not need to satisfy the (c) criterion to be included, as we were interested in qualitative definitions of self-determination/autonomy, control, and autonomous reliance. Studies were excluded if residents were only in temporary care (e.g., stints in hospital). We did not limit studies by date or by language. As a result, we translated two French-language papers to English prior to data extraction, using Google Translate.

### Information Sources

We searched the following databases for relevant articles: Academic Search Complete, ERIC, MEDLINE Complete, PsycArticles, PsycExtra, Psychology and Behavioral Sciences Collection, PsycInfo, Open Dissertations, Scopus, Web of Science, and CINAHL Complete. The search was completed on May 19, 2022. We report the search terms in Supplementary Material S1.

### Abstract and Full-Text Screening

**Figure 2.**
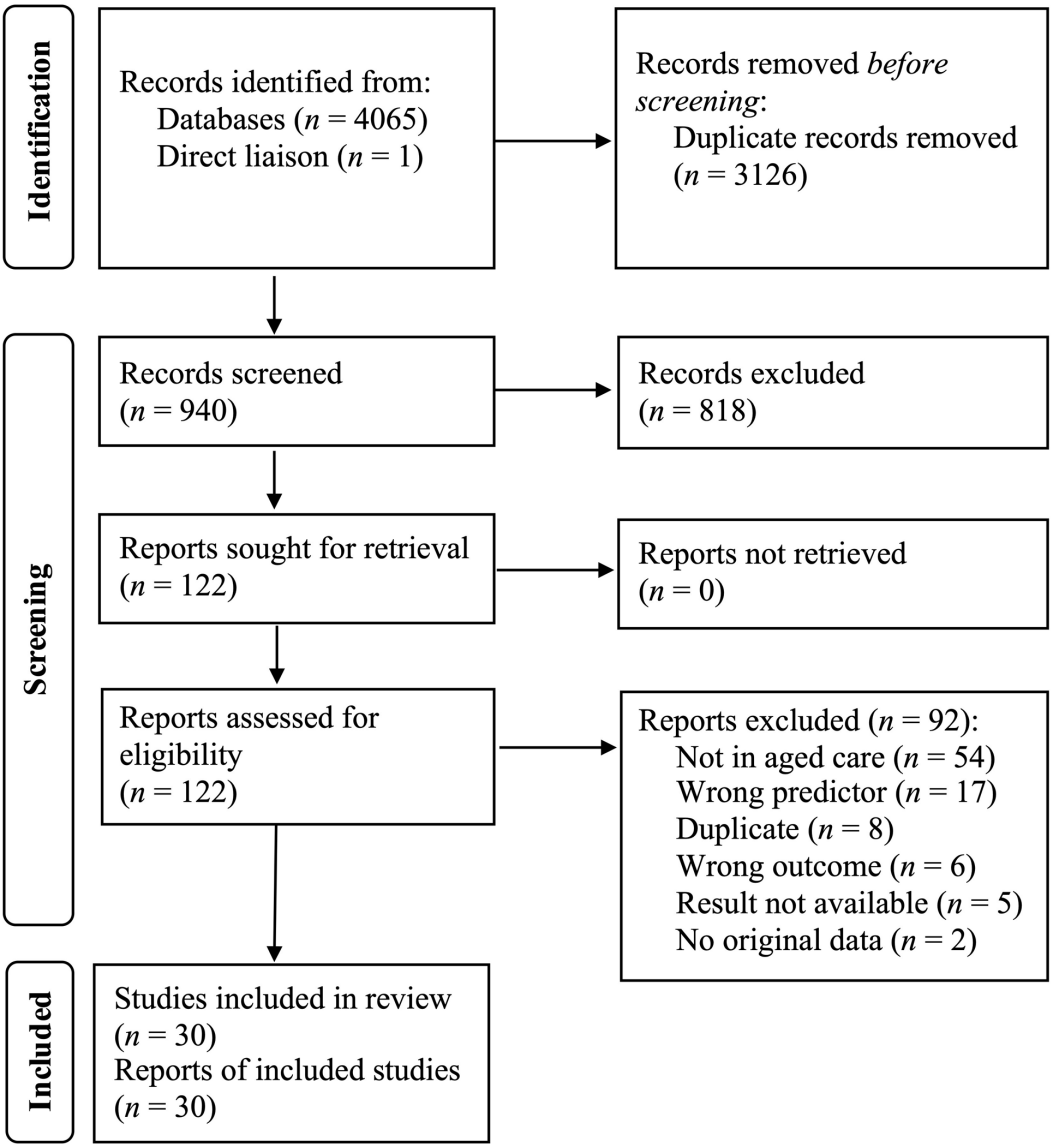
The flow of studies using the Preferred Reporting Items for Systematic Review and Meta-Analyses (PRISMA; Page et al., 2021) diagram.

We used the Covidence software to screen abstracts and full texts. Two coauthors independently screened each abstract and full text. Any disagreements were resolved by negotiation between the two screeners. Figure 2 shows the Preferred Reporting for Systematic Reviews and Meta-Analyses (PRISMA, Page et al., 2021) flow diagram of reports through the screening process. The full PRISMA systematic review checklist is available in Supplementary Material S2. We detail the subsequent stages of the review (i.e., data extraction—including effect size/moderator coding) in Supplementary Material S3. We evaluated quantitative study quality using a four-point system borrowed from recent SDT meta-analyses (Bradshaw et al., 2022) and qualitative study quality using the 10-item Critical Appraisal Skills Program (CASP, 2013). Both methods are detailed in Supplementary Material S4. Summaries of the included studies and their characteristics are available in Supplementary Materials S5 and S6.

### Analytic Strategy

#### Quantitative

All analyses were performed using R version 4.1.1 (2021-08-10) (R Core Team, 2021). The *metafor* package was used to calculate effect sizes (Viechtbauer, 2010). Pearson’s *r* correlations were extracted and transformed to Fisher’s *z* for analysis. Following modeling, results were back transformed to *r* to enhance interpretability. We evaluate effect size according to the thresholds proposed by Gignac and Szodorai (2016) (i.e., *r* = 0.10, 0.20, and 0.30, signify relatively small, typical, and relatively large effects, respectively). We used “StudyID” (i.e., report identification number) to cluster our data, meaning that when multiple effects were extracted from the same report, we accounted for their dependency. We used the R package *metaSEM* (Cheung, 2015b) to conduct three-level meta-analytic structural equation models (Cheung, 2015a). In these models, the first level pools effect sizes at the participant level. The second and third levels model within (τ²₍₂₎) and between (τ²₍_3_₎) study heterogeneity, respectively. For the autonomy and control models, we report the pooled effect sizes, 95% confidence intervals, and the amount of heterogeneity within (level 2) and between (level 3) studies.

#### Qualitative

The aim of our qualitative meta-synthesis was to understand how autonomy, control, and autonomous reliance are experienced in residential aged care from the perspectives of residents. We used a grounded theory approach in our meta-synthesis. Grounded theory meta-synthesis is an iterative approach that involves coding data from primary studies using NVivo, creating code phrases, reducing them into clusters, and developing categories and subcategories (Eaves, 2001). This approach has gained traction in meta-synthesis because it allows primary data to be integrated, allowing novel theoretical insights that can guide future research (Eaves, 2001). The method also permits in-depth summaries of relevant research without the dependence on very large numbers of studies (Bilsen & Hannes, 2014). Consistent with the methods developed by AlOmeir et al. (2020), we meta-synthesized our data using a combination of classic (Glaser & Strauss, 2017) and Straussian (Strauss & Corbin, 1990) grounded theory methods with an interpretivist approach.

Given that SDT was our theoretical framework, we anchored our grounded theory meta-synthesis using “autonomy”/“self-determination,” “control,” and frequently appearing synonyms for “autonomous reliance” (see Supplementary Material S2) as “sensitizing concepts” (Bowen, 2006, p. 12). Sensitizing concepts provide a guide to help grounded theory researchers identify specific examples of phenomena (Bowen, 2006). As per AlOmeir et al.’s (2020) method, data comprised “participant quotes evidenced within the original studies” (p. 5077) extracted from the full-text records and uploaded to NVivo for thematic coding, following the basic premises of thematic analysis (Braun & Clarke, 2006). Our primary foci were residents’ verbatim definitions and descriptions; however, we supplemented residents’ quotes with researchers’ quotes if they were based on analyses of residents’ data. Data for the three sensitizing concepts were analyzed separately, according to the method outlined by AlOmeir et al. (2020). First, we collaboratively pooled the data into first-order constructs using open coding by selecting notable and consistent words or phrases that eventually represented all the extracted data, we then used axial coding to sort the open codes into groups depending on whether they were similar or differentiable in a meaningful way, we then reviewed the axial codes and used selective coding, iterating back and forth to the data, to link related codes under distinct themes.

## Results

### Reports and Participants

From an initial pool of 940, and based on the above-mentioned inclusion criteria, we identified 30 eligible reports (19 studies with quantitative effects and 11 studies including qualitative data) spanning 28 years (from 1994 to 2022). These studies contained 141 quantitative effects and 84 pieces of relevant qualitative data. The total number of participants was 2,668 (calculated using the largest sample size reported per manuscript). Most studies were conducted in France (25.81%), followed by Belgium (12.90%), Sweden (9.68%), and Canada (9.68%).

### Systematic Review and Evidence Gaps

**Figure 3.**
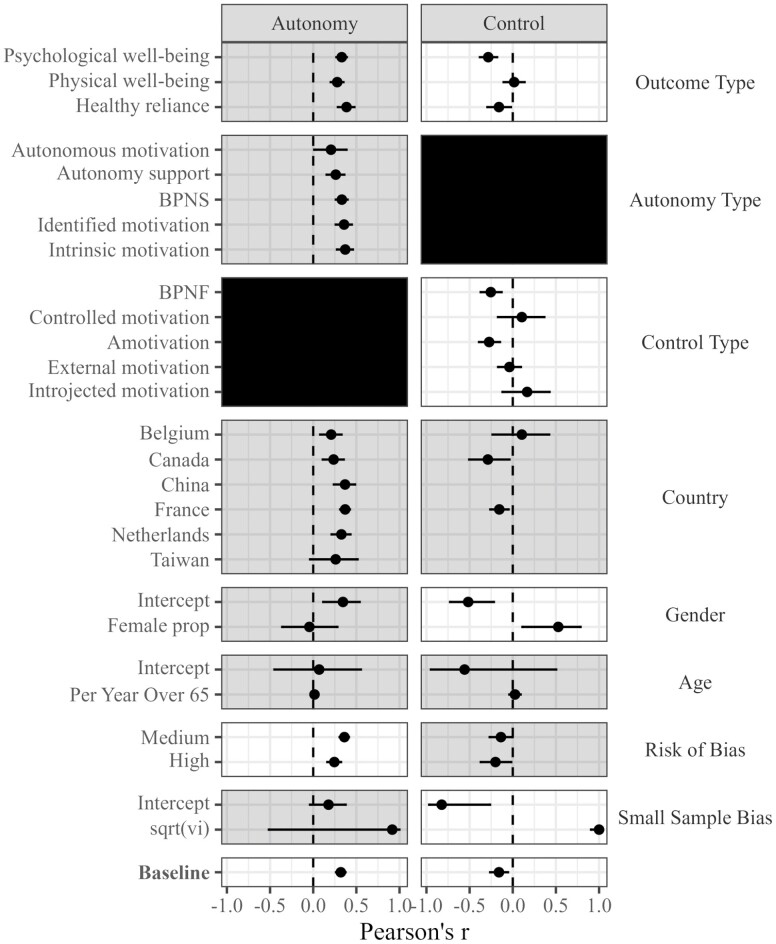
Moderation matrix of the effects of covariates across the autonomy and control meta-analytic models based on the 18 quantitative studies included in the analysis. Empty rows represent an absence of data. White and gray cells represent moderators that were and were not statistically significant, respectively. Black cells are not relevant to the model in that column. Intercept = the baseline model estimated where the covariate (i.e., gender, age, small sample bias) is equal to zero, female prop = a continuous variable indicating an increasing proportion of females, age = mean age of the samples minus 65, such that it assesses change in the links per year of life since 65, Sqrt(vi) = the square root of the variance, baseline = the model without any adjustment for moderating covariates, BPNS = basic psychological need satisfaction, BPNF = basic psychological need frustration.

In addition to pooling data and effects, a systematic review should first summarize the literature to identify evidence “gaps.” Evidence gaps refer to demographic and/or methodological areas that have not been studied or have been understudied with reference to the variables of interest. Knowledge about the breadth of available data informs the reliability and generalizability of the meta-results. Figure 3 serves as a “map” of potential evidence gaps in the evidence base by providing an at-a-glance summary of the data available at various levels of theoretically and methodologically relevant potential moderators. Figure 3 depicts the baseline effects of experiences of autonomy and being controlled on aged care residents’ optimal functioning, as well as the availability of effects by specific moderators (e.g., country of origin, proportion of females in the samples, and so on). White and gray cells indicate statistically significant and nonsignificant moderators, respectively. Empty cells represent an absence of data.

It is clear from the patterns of statistically significant and nonsignificant moderators shown in Figure 3 that the effects for residents’ experiences of autonomy were more consistent than they were for control. Not clear from the evidence gap map, though notable, is the fact that 54 studies were omitted due to not being conducted in residential aged care facilities (see Figure 3). Clearly, there is research interest in older adults’ experiences of autonomy and control, however, the relative lack of studies in residential aged care facilities coupled with aged care residents’ lower degree of wellbeing (compared to the general community) suggests that more context-specific work is needed. Also clear from the systematic review is a lack of longitudinal studies. The pooled effects in the meta-analysis represent only correlations. Thus, the causal or temporal relations cannot be surmised.

Randomized controlled trials (RCTs) are considered the gold standard for establishing causation. Our systematic review found two RCTs (Buckinx et al., 2020; Davison et al., 2022), which are too few to meta-analyze. Pooling of the effects from these two studies would have been especially problematic because, while they shared theoretical underpinnings, the experimental methods were heterogeneous. Nonetheless, both studies reported that interventions structured around providing experiences of autonomy reduced residents’ anxiety (Davison et al., 2022) and improved their physical activity (Buckinx et al., 2020).

### Quantitative Meta-Analyses

#### Residents’ experiences of autonomy and their wellbeing outcomes

**Figure 4.**
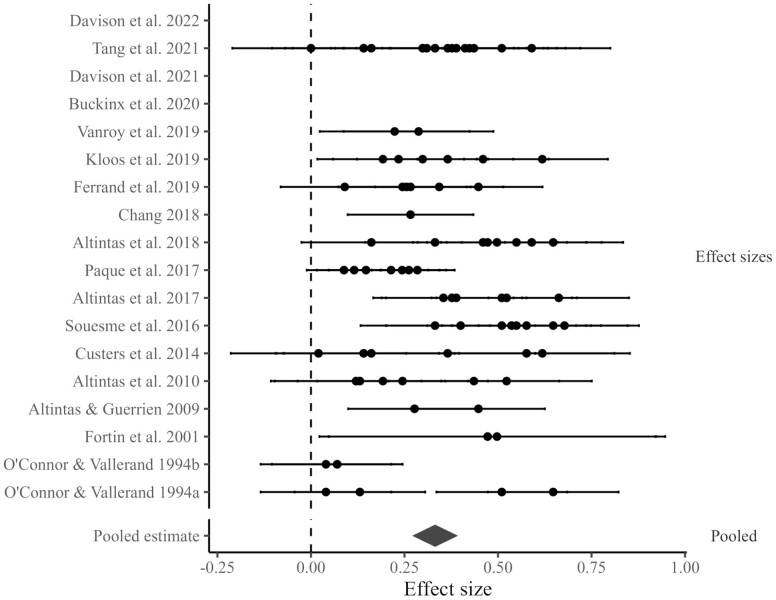
A forest plot of the effects linking residents’ experiences of autonomy with their wellness outcomes.

As shown in Table 1 and Figure 4, 18 studies (including 85 effect sizes) reported data that could be pooled. There was a statistically significant, relatively large, positive pooled effect of aged care residents’ experiences of autonomy and their wellbeing outcomes, *r* = 0.33 [95% CI 0.27, 0.39]. Inspection of the *Q* statistic revealed statistically significant heterogeneity *Q*(84) = 310.01, *p* < .001. The heterogeneity at level 2 (within-study) was 42.78%. The heterogeneity at level 3 (between-studies) was 31.19%.

**Table 1.** Meta-Regressions for the Pooled Link Between Aged Care Residents’ Experiences of Autonomy and Their Wellness Outcomes, and the Assessment of Seven Possible Moderators of the Pooled Effect

Moderation	*k*	*n*	*r* [95% CI]	*z*	*SE*	*p*	$\tau_{\left(2\right)}^{2}$	$\tau_{\left(3\right)}^{2}$	$R_{\left(2\right)}^{2}$	$R_{\left(3\right)}^{2}$	Likelihood ratio test
Baseline	18	85	0.33 [0.26, 0.37]	0.33	0.03	<.001	0.01	0.01			
Outcome type	18	85					0.01	0.01	5.73	17.93	$\chi^{2}$ (2) = 4.10, p = .13
Psychological wellbeing	15	65	0.33 [0.27, 0.39]	0.34	0.03	<.001					
Physical wellbeing	9	25	0.28 [0.20, 0.35]	0.29	0.04	<.001					
Autonomous reliance	5	13	0.39 [0.29, 0.48]	0.41	0.06	<.001					
Autonomy type	18	85					0.01	0.01	0.00	41.47	$\chi^{2}$ (4) = 3.25, p = .52
Autonomous motivation	2	3	0.21 [0.01, 0.39]	0.21	0.10	.038					
Autonomy support	8	32	0.26 [0.16, 0.36]	0.27	0.06	<.001					
BPNS	6	44	0.33 [0.26, 0.40]	0.35	0.04	<.001					
Identified motivation	5	12	0.36 [0.26, 0.45]	0.37	0.05	<.001					
Intrinsic motivation	5	12	0.37 [0.28, 0.46]	0.39	0.05	<.001					
Country	18	85					0.01	0.00	0.81	57.10	$\chi^{2}$ (5) = 6.31, p = .28
Belgium	3	12	0.21 [0.08, 0.33]	0.21	0.07	.001					
Canada	3	9	0.24 [0.11, 0.35]	0.24	0.07	<.001					
China	1	9	0.37 [0.24, 0.49]	0.39	0.07	<.001					
France	7	45	0.37 [0.31, 0.42]	0.39	0.03	<.001					
Netherlands	2	12	0.33 [0.21, 0.43]	0.34	0.06	<.001					
Taiwan	1	1	0.26 [−0.04, 0.51]	0.27	0.15	.084					
Gender	17	83					0.01	0.01	0.40	0.00	$\chi^{2}$ (1) = 0.07, p = .79
Intercept			0.34 [0.12, 0.54]	0.36	0.12	.004					
Female prop			−0.04 [−0.36, 0.28]	−0.04	0.17	.79					
Age	18	85					0.01	0.01	0.00	10.61	$\chi^{2}$ (1) = 0.84, p = .36
Intercept			0.07 [−0.45, 0.55]	0.07	0.28	.81					
Per year over 65			0.02 [−0.02, 0.05]	0.02	0.02	.35					
Risk of bias	18	85					0.01	0.00	0.00	50.63	$\chi^{2}$ (1) = 4.36, p = .037
Medium	11	76	0.36 [0.31, 0.41]	0.38	0.03	<.001					
High	7	27	0.24 [0.16, 0.32]	0.25	0.04	<.001					
Small sample bias	18	85					0.01	0.01	0.58	15.96	$\chi^{2}$ (1) = 1.97, p = .16
Intercept			0.18 [−0.04, 0.38]	0.18	0.11	.10					
√ variance			0.92 [−0.51, 1.00]	1.56	1.09	.15					

*Notes*: *k* = number of studies, *n* = number of effects. *r* = Pearson’s correlation, *z* = Fisher’s *z* transformed correlation, *SE* = standard error of Fisher’s *z* transformed correlation, *p* = *p* value of each slope, *R*^2^_(2)_ = % of within study heterogeneity explained by the model, *R*^2^_(3)_ = % of between study heterogeneity explained by the model, Likelihood ratio test = tests if the model that includes the moderator is an improvement over the baseline model, BPNS = basic psychological need satisfaction, intercept for gender = the baseline model estimated where the % of females in the sample is zero, female prop = a continuous variable indicating an increasing proportion of females, intercept for age = the baseline model estimated where the average age is 65, per year over 65 = years of age beyond 65 as a continuous variable.

There were no statistically significant demographic or methodological moderators. The strong positive effect of autonomy applied regardless of the type of wellbeing outcome, how autonomy was indexed, the country of origin, the proportion of females in the sample, or residents’ age. We did detect moderation by “Risk of Bias,” though interestingly, we found that studies with a higher risk of bias had smaller effects than studies of only moderate risk of bias. Effects at both levels of risk were substantial and positive, so moderation by “Risk of Bias” should not be seen to undermine the thrust of our results. There was no evidence of publication bias in this model, $\chi^{2}$(1) = 1.97, *p* = .16. Visual inspection of the funnel plot (Supplementary Material S7) showed no asymmetry.

#### Residents’ experiences of control and their wellbeing outcomes

**Figure 5.**
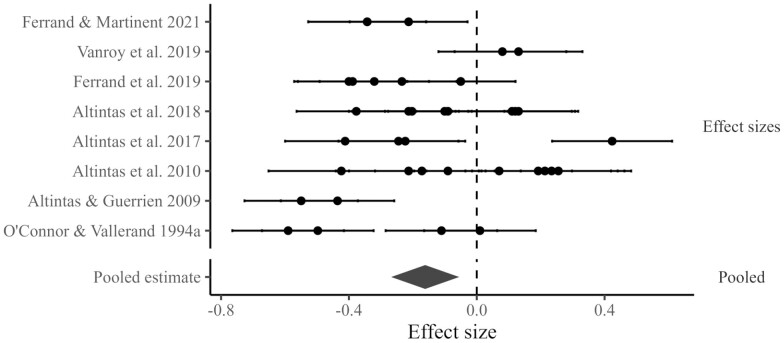
A forest plot of the effects linking residents’ experiences of control with their wellness outcomes.

As shown in Table 2 and Figure 5, eight studies (including 38 effect sizes) reported data that could be pooled. There was a statistically significant, relatively small, negative pooled effect of aged care residents’ experiences of control and their wellbeing outcomes, *r* = −0.16 [95% CI −0.27, −0.06]. Inspection of the *Q* statistic revealed statistically significant heterogeneity *Q*(37) = 260.92, *p* < .001. The heterogeneity at level 2 (within-study) was 69.63%. The heterogeneity at level 3 (between studies) was 17.43%. The covariates that statistically significantly moderated the baseline model were outcome type, control type, and gender.

**Table 2.** Meta-Regressions for the Pooled Link Between Aged Care Residents’ Experiences of Control and Their Wellness Outcomes, and the Assessment of Seven Possible Moderators of the Pooled Effect

Moderation	*k*	*n*	*r* [95% CI]	*z*	*SE*	p	$\tau_{\left(2\right)}^{2}$	$\tau_{\left(3\right)}^{2}$	$R_{\left(2\right)}^{2}$	$R_{\left(3\right)}^{2}$	Likelihood ratio test
Baseline	8	38	−0.16 [−0.26, −0.05]	−0.16	0.05	.003	0.04	0.01			
Outcome type	8	38					0.03	0.00	29.38	71.19	$\chi^{2}$ (2) = 12.52, p = .002
Psychological wellbeing	6	15	−0.28 [−0.38, −0.18]	−0.29	0.06	<.001					
Physical wellbeing	5	13	0.02 [−0.11, 0.14]	0.02	0.06	.80					
Autonomous reliance	4	10	−0.16 [−0.29, −0.02]	−0.16	0.07	.025					
Control type	8	38					0.03	0.00	31.35	96.03	$\chi^{2}$ (4) = 16.68, p = .002
BPNF	2	9	−0.25 [−0.37, −0.13]	−0.26	0.07	<.001					
Amotivation	5	12	−0.27 [−0.39, −0.15]	−0.28	0.07	<.001					
Controlled motivation	1	2	0.11 [−0.17, 0.37]	0.11	0.14	.46					
External motivation	5	12	−0.04 [−0.17, 0.09]	−0.04	0.07	.57					
Introjected motivation	1	3	0.17 [−0.12, 0.43]	0.17	0.15	.25					
Country	8	38					0.04	0.01	3.51	49.01	$\chi^{2}$ (2) = 3.05, p = .22
Belgium	1	2	0.11 [−0.23, 0.42]	0.11	0.18	.55					
Canada	1	4	−0.29 [−0.50, −0.04]	−0.30	0.13	.024					
France	6	32	−0.16 [−0.26, −0.05]	−0.16	0.06	.004					
Gender	8	38					0.04	0.00	1.11	90.14	$\chi^{2}$ (1) = 4.64, p = .031
Intercept			−0.52 [−0.73, −0.22]	−0.57	0.18	.001					
Female prop			0.53 [0.11, 0.79]	0.59	0.24	.015					
Age	8	38					0.04	0.01	0.00	17.46	$\chi^{2}$ (1) = 0.59, p = .44
Intercept			−0.56 [−0.95, 0.50]	−0.63	0.60	.30					
Per year over 65			0.03 [−0.04, 0.09]	0.03	0.03	.43					
Risk of bias	8	38					0.04	0.01	2.75	0.00	$\chi^{2}$ (1) = 0.30, p = .58
Medium	5	23	−0.14 [−0.27, −0.01]	−0.14	0.07	.042					
High	3	15	−0.20 [−0.37, −0.02]	−0.20	0.09	.032					
Small sample bias	8	38					0.04	0.00	5.35	100.00	$\chi^{2}$ (1) = 6.77, p = .009
Intercept			−0.82 [−0.97, −0.26]	−1.17	0.46	.011					
√ variance			1.00 [0.91, 1.00]	10.4	4.55	.022					

*Notes: k* = number of studies, *n* = number of effects, *r* = Pearson’s correlation, *z* = Fisher’s *z* transformed correlation, *SE* = standard error of Fisher’s *z* transformed correlation, *p* = *p* value of each slope, *R*^2^_(2)_ = % of within study heterogeneity explained by the model, *R*^2^_(3)_ = % of between study heterogeneity explained by the model, Likelihood ratio test = tests if the model that includes the moderator is an improvement over the baseline model, BPNF = basic psychological need frustration, intercept for gender = the baseline model estimated where the % of females in the sample is zero, female prop = a continuous variable indicating an increasing proportion of females, intercept for age = the baseline model estimated where the average age is 65, per year over 65 = years of age beyond 65 as a continuous variable.

Moderation by outcome type indicated that experiences of control are especially costly to aged care residents’ psychological wellbeing and autonomous reliance. There was no effect of control on residents’ physical wellbeing. Moderation by control type indicated that residents’ reports of basic psychological need frustration and amotivation were linked negatively with their wellbeing outcomes, whereas the link with controlled forms of motivation was not statistically significant. Moderation by gender indicated that men suffer more than women under controlling conditions. The slope for female proportion represents the effect for samples comprising only females. To interpret the slope, it is summed with the intercept, meaning that, in these data the effect of control on residents’ wellbeing outcomes in female-only samples is equivalent to zero. The more women that comprise the samples the smaller the negative effect of control on wellbeing outcomes.

In these data, there was evidence of statistically significant publication bias, $\chi^{2}$(1) = 5.48, *p* = .019. The standard errors were positively correlated with the effect sizes indicating that under conditions of high uncertainty, effect sizes were stronger than would otherwise be expected. However, we inspected the funnel plot (Supplementary Material S8) and found no discernible pattern of bias, so while this result should be noted, it may be artifactual.

### Qualitative Meta-Synthesis

Of the 84 pieces of qualitative data identified, *n* = 39 pertained to autonomy/self-determination, *n* = 27 summarized experiences of control, and *n* = 18 captured perspectives on autonomous reliance.

#### Experiences of autonomy in aged care

Two key themes emerged from the published data examining residents’ descriptions and definitions of autonomy: *involvement in decision-making* and *an individuated approach.* Both themes revealed specificity and heterogeneity about what was important to residents.

##### Involvement in decision-making

The importance of decisional control was evident in residents’ quotes such as: “… making my own decisions is a want and a need, it’s just me,” “I would like to be in charge of my life and do what I choose to do” (Macleod, 2018, p. 101), and “I decide over my own schedule… and that is a good feeling” (Nakrem et al., 2011, p. 1362). These quotes are consistent with questions from typical self-report measures of autonomy satisfaction (e.g., I feel a sense of choice and freedom in the things I undertake, from Chen et al., 2015). Three decision-making subthemes revealed that aged care residents’ experiences of autonomy can be bolstered in three specific ways: *daily living*, *treatment*, and *entering aged care*.

The daily living subtheme indicated that aged care residents want to feel in control of everyday tasks and decisions. Common were desires “make my bed myself” (Bollig et al., 2016, p. 147), “decide what I like to eat” (Macleod, 2018, p. 120), and “arrange my closet” (Macleod, 2018, p. 109), also frequent were references to in-moment ­decision-making such as whether to have company (e.g., “…they shall knock on the door,” Bollig et al., 2016, p. 148). The two other subthemes—treatment and entering aged care—were important though less prominent in the data. As examples, Schenell et al. (2020) reported that residents’ “feeling of control was strengthened when they could decide how care should be provided” (p. 152), and Andersson et al. (2007) identified that all self-reported “satisfied” (p. 1713) residents had been involved in the decision to move into residential care.

##### Individuated approach

The importance of being treated as an individual was also central to aged care residents’ experiences of autonomy. Supporting data referred both to the residents’ desires (e.g., “I do not want to be one in an anonymous gray mass, I want to stand out, so to speak,” Schenell et al., 2020, p. 153), as well as researchers’ awareness that an individuated approach would be beneficial to residents’ wellbeing (e.g., “… to enhance self-determination, the staff could help the resident understand that certain areas could be controlled by the residents themselves,” Bollig et al., 2016, p. 150, and “[Residents want to] live in [their] own way,” Nakrem et al., 2011, p. 1362). Residents’ desires for an individuated approach are consistent with SDT’s conceptualization of autonomy support, which is an adaptive approach, not something one-size-fits-all.

#### Experiences of control in aged care

Three key themes emerged from the published data examining residents’ descriptions and definitions of control: *constantly controlled*, *being overlooked*, and *staff rule, staff cruel.*

##### Constantly controlled

Residents reported feeling constantly controlled. For example, they reported “being controlled around the clock” (Bollig et al., 2016, p. 148) and were not permitted to make everyday decisions of which they were capable, as evidenced in this passage: “participants described that they were forced to follow rules that applied to all residents, regardless of their capabilities” (Schenell et al., 2020, p. 150). Residents’ examples of feeling constantly controlled included “If you want to go to the toilet, they say ‘you just went a couple of hours ago’, so that’s that” (Hellström & Sarvimäki, 2007, p. 419) and “some residents have to go to bed at six o’clock in the evening” (Bollig et al., 2016, p. 148).

##### Being overlooked

Residents also experienced control when they felt overlooked, especially when it came to decision-making, saying things like “I can’t decide anything for myself. I don’t have any influence…” (Hellström & Sarvimäki, 2007, p. 420) and researchers reported that “residents were neither included in any decision-making processes, nor were they asked for their opinion” (Kaelen et al., 2021, p. 9).

##### Staff rule, staff cruel

Residents felt controlled when staff prioritized their own rules and routines over residents’ needs and wants, “the staff wanted to rule and staff routines directed daily living” and reported feeling that “it has to go according to their rules” (Hellström & Sarvimäki, 2007, p. 419). Some residents acknowledged that the focus on staff schedules was a function of inadequate staffing, but others felt controlled by staff cruelty, “Sometimes they are snapped at, the old ones. Because they can’t do everything, or they don’t do it fast enough” (Schenell et al., 2020, p. 152). In some instances, residents experienced the cruelty as dehumanizing: “They don’t tell you anything at all, but they informed my daughter, or other people from the family, but you... who are you? Nothing.” (Kaelen et al., 2021, p. 9). Researchers reported that residents’ felt controlled by this type of treatment because residents “must accept the staff’s treatment as they were dependent on them and were afraid of being disliked or seen as whiners” (Schenell et al., 2020, p. 151).

#### Experiences of autonomous reliance in aged care

The importance of an autonomous ceding of select responsibilities was evident in these data. Researchers reported that residents “did not mind being cared for in certain areas they have perceived as areas they have willingly chosen to give up, such as in the routine of preparing and being served food” (MacLeod, 2018, p. 144), a perspective also endorsed by residents, “I wouldn’t have been able to manage on my own any more, and I didn’t want to” (Schenk et al., 2013, p. 2934), and “I think differently [about being in the nursing home] than I did in the beginning when I came here... because I now feel more connected [to the nursing home] than I did when I came here. I do feel more at home” (Bollig et al., 2016, p. 146). Two specific themes emerged as representing residents’ experiences of autonomous reliance in the aged care context: *safety in reliance* and *the role of staff*.

##### Safety in reliance

When reliance is autonomous, these data suggest that it can serve as a source of comfort and safety, “of course I am dependent. And that is a feeling of safety” (Nakrem et al., 2011, p. 1362), “there’s a certain feeling of security here” (Schenk et al., 2013, p. 2935), and having staff “available around the clock also provided feelings of gratitude, peace, and increased security” (Schenell et al., 2020, p. 153).

##### The role of staff

Perhaps unexpectedly, residents reported that their ability to autonomously rely on the staff “depends on how the nurses are” (Bollig et al., 2016, p. 147). Staff behaviors including body language (e.g., “Their way to behave, their face… counts very much,” Bollig et al., 2016, p. 147), verbal communication, and interest (e.g., “residents tried to find opportunities to tell staff about who they were and what they had achieved earlier in life,” Schenell et al., 2020, p. 153) were factors underpinning their abilities to provide individuated care (e.g., “staff who adjusted the care to the residents’ needs,” Schenell et al., 2020, p. 153) and build trust with the residents (e.g., “good nurses built trust-based relationships with the residents,” Bollig et al., 2016, p. 150).

## Discussion and Implications

The quantitative results from our mixed-methods review showed that experiences of autonomy and control, respectively helped (Hypothesis 1) and hindered (Hypothesis 2) aged care residents’ physical and psychological wellbeing and their autonomous reliance on aged care staff. If the wellbeing of aged care residents is a priority—which we think it simply must be—these results suggest that the sector should consider policies designed to ensure aged care residents receive appropriate autonomy support, as well as reforms to ameliorate the psychological harms associated with even the necessary use of control. To inform the possible specifics of such policies, the qualitative results from our meta-synthesis also revealed seven primary and three sub-themes that describe the nuanced ways autonomy, control, and autonomous reliance are manifest in the lives of aged care residents. Below, we discuss the implications of the quantitative results, by integrating their import with the revelations from the qualitative analysis.

### The Universal Importance of Autonomy

Consistent with our SDT-based predictions, the positive effect of autonomy on aged care residents’ wellbeing was consistent across types of wellbeing (Hypothesis 3) and experiences of autonomy (Hypothesis 4), and applied across countries, resident age, and the gender composition of the samples (Hypothesis 6). Evidently, the social structures and resources designed to support aged care residents’ wellbeing should bolster their autonomy in addition to more practical measures.

Aged care residents’ readiness to rely on staff—which is fundamental to their optimal functioning in nursing home life—was a particularly strong outcome of their experiences of autonomy. Erikson et al. (1986) proposed that during older adulthood, humans reexperience the autonomy versus shame and doubt stage of psychosocial development. Older adults need to balance their previous self-reliance with their emerging cognitive and physical limitations (Nusbaum, 2010). Erikson et al. (1986) found that a sense of acceptance about one’s limits facilitated feelings of autonomy and autonomous help-seeking. However, if one experienced shame about their impairments, they experienced less autonomy and sought less help. Clearly, aged care staff can play a pivotal role in shaping residents’ feelings about their limitations. If staff induce residents’ shame about their abilities—as was evidenced in the qualitative theme of *staff rule, staff cruel*—their perceived autonomy would be undermined along with their wellbeing and willingness to seek help (Nusbaum, 2010). In contrast, residents could experience more autonomous reliance if staff provide autonomy support, in the forms of *involvement in decision-making* and *an individuated approach,* as we showed in the results of the meta-synthesis.

### The Nuanced Effects of Control in Aged Care

Experiences of being controlled in residential aged care appear detrimental to wellbeing. We saw evidence of this at both the quantitative and qualitative levels. Residents described feeling degraded, dehumanized, and overlooked. Thus, the negative effect of control on residents’ wellbeing is understandable. However, the magnitude of the effect is somewhat smaller than that of autonomy. The presence of multiple moderators also suggests that the effects of control are more nuanced than they are for autonomy. Our analysis pooled different types of control: basic psychological need frustration, amotivation, and external and introjected forms of motivation. Of these types, basic psychological need frustration and amotivation had the strongest negative links with aged care residents’ wellbeing. External and introjected motivations were not linked with wellbeing. These differentiated effects are consistent with evidence that amotivation, external motivation, and introjected motivation link to adaptive outcomes in a graded way (Donald et al., 2020; Vasconcellos et al., 2020). Amotivation is almost universally costly, external motivation tends to be costly but less so, and introjection can be only weakly, negatively linked—and often *not* linked—with adaptive outcomes. Our results are further evidence of this previously demonstrated progressive pattern of associations.

The graded associations between controlled forms of motivation and outcomes may be particularly relevant to physical health (Vasconcellos et al., 2020). Although amotivation has been shown to undermine physical activity and other adaptive outcomes, external and introjected motivations are still *motivational*. Particularly in the domain of physical activity, external and introjected motivations *can* spur action, just not reliably or over the long-term. The differentiated effects of different types of controlled motivation could speak to our finding that, while control undermined psychological wellbeing and autonomous reliance, it was not linked with physical wellbeing.

Some older adults may be motivated to be physically active to avoid guilt (i.e., introjected motivation), others by rewards from staff for exercising (i.e., external motivation). These motives can benefit the short-term physical wellbeing of some but not others. Indeed, the confidence intervals for the links between external and introjected motivations and physical wellbeing include zero, meaning that the effects are (a) not statistically significant overall and (b) positive for some and negative for others. By pooling the negative effects of amotivation and need frustration with the inconsistent effects of external and introjected motivation, the overall effect of control on physical wellbeing was likely reduced to zero. It is important to note, however, that external and introjected motivations did not *positively* link with physical wellbeing. Therefore, to promote long-term, sustained, physical wellbeing among aged care residents, autonomy support should still be prioritized over any specific controlling strategies.

#### The role of gender and control

Our quantitative results indicate that conditions of control predict more male than female suffering in residential aged care. This result is consistent with previous mixed-methods evidence that men experience lower quality of life in aged care in general (Davila et al., 2022). Entering aged care necessitates the ceding of responsibility, and men may be particularly vulnerable to subsequent feelings of disempowerment and a loss of social status (Jilek, 2006). For samples of predominantly females, we found that control was not linked to wellbeing. However, these results for gender should not be interpreted as meaning women benefit from control. Instead, the results for gender and control indicate that gender norms and stereotypes could be important considerations in the development and implementation of policy and procedure that may make residents more prone to experiencing the costs of control.

### Qualitative Experiences of Autonomy, Control, and Reliance

The themes derived from the qualitative meta-synthesis shed nuanced light on how aged care residents’ experience autonomy, control, and reliance on others. These results are intended to inform the “how” of providing autonomy support to aged care residents, though we acknowledge that the specifics of that application will certainly vary depending on residents’ physical and cognitive capabilities and the type of care they need. Nonetheless, the emergent themes spoke to several general principles regarding autonomy support in aged care. The themes indicated that many aged care residents feel constantly controlled and, as a result, feel their wellbeing is undermined. Instead, and unsurprisingly, residents want to have opportunities for choice and to feel like individuals, and when they do, they feel supported and safe.

### Implications for Policy and Procedure

By including residents’ own voices, we have been able to achieve a level of specificity that could uniquely inform aged care policy and procedure. For example, aged care residents often accept their waning capabilities, and thus expect their choice points to be more menial. They would like to decide what to eat, when to shower, and what time to sleep, among other everyday things. Possibly, residential procedures could be developed to ensure that some of the daily one-on-one time allocated to residents could be spent making individualized choice-based plans. Similarly, because microexperiences such as facial expression and tone of voice were factors in residents’ experiences of control and readiness to seek help, policies could be established to ensure that staff engage in ongoing nonverbal communication skills training to enhance their autonomy support. Indeed, recent SDT research has demonstrated that nonverbal communication—such as effective listening—is an important element of caring relationships (Weinstein et al., 2022). Future research should examine these possibilities and explore experiences of autonomy and control in community-dwelling older adults to see how they relate to the experiences of aged care residents.

It is important to acknowledge that enhancements to autonomy-supportive care will place additional demands on an already stretched aged care workforce. Therefore, policies designed to allow staff more discretion over their time need to be considered alongside efforts to improve the autonomy support they provide. Ideally, more staff would be rostered to provide better scope for individuated care. Though, there is a known workforce shortage that needs to be understood via future research. Why are aged care staff leaving the sector? This question needs answering because staff are fundamental to the provision of quality care. There is evidence in other sectors such as education, that when staff are they themselves controlled (e.g., by supervisors and/or by controlling institutional policies and procedures) they experience less job commitment, *and* they treat those in their charge more controllingly (Reeve, 2009). In contrast, when staff receive autonomy support, they provide more autonomy support to others. Therefore, amending the framing and delivery of institutional aged care policies to better support the autonomy of staff might naturally increase their provision of autonomy support and therefore the wellbeing of residents. These possibilities should be tested in residential aged care and, if supported, a review of policies and management styles could improve conditions for both staff and residents.

### Limitations

The literature under review is limited in three primary ways. First, there is an absence of longitudinal or experimental evidence, which means only noncausal interpretations can be made. SDT-based residential aged care research would be furthered by the development of interventions designed to improve residents’ wellbeing via autonomy support. Past SDT research has demonstrated the effectiveness and benefits of autonomy support interventions in schools (Cheon et al., 2019), healthcare (Williams et al., 2000), sports (Cheon et al., 2015), workplaces (Slemp et al., 2018), and families (Joussemet et al., 2008), so there is good reason to expect that such an intervention would prove useful for staff and residents in aged care.

Second, the literature is western-centric, with most of the data coming from Caucasian-majority European countries. Although this does not undermine the validity of the findings, it does call into question their cross-cultural generalizability. Finally, there was some evidence of a small sample bias in the control model of the meta-analysis. This is likely driven by data collection limits; specifically aged care samples are generally small and often heterogeneous.

There are also limitations of this review itself. Despite searching the gray literature, our results revealed no unpublished quantitative studies. Given only published articles were meta-analyzed, the effects could be inflated. In addition, when extracting the qualitative data, we used a combination of verbatim quotes from aged care residents and descriptions synthesized by the source authors. As with any meta-synthesis of qualitative data, we were only able to synthesize data that the source authors had selected. As such, we acknowledge that any bias introduced by source authors is being invertedly carried forward in this review.

## Concluding Remarks

The results from this systematic review indicate that autonomy appears to be a fundamental piece of the puzzle in terms of supporting the wellbeing of adults in aged care. We have also revealed a clearer picture of what experiences of autonomy, control, and autonomous reliance look like from those who matter most in this context: the residents. The results were consistent with our SDT-based hypotheses and with the principles of “person-centered” practice, which is care based on the person’s preferences, needs, and values (Edvardsson, 2015). Indeed, much of SDT’s definition of autonomy support is consistent with the specific principles of person-centered practice. For example, autonomy support involves making people feel heard and understood, as well as respected and nurtured, which is consistent with the principles of dignity and voice within the person-centered care model (Kwame & Petrucka, 2021). Although future research should examine more closely the specific elements and mechanisms of autonomy support and person-centered care, we hope that the results of this meta-analysis are heeded by the aged care industry in the development of policy. Or results suggest that policy makers should bring autonomy support into focus and take steps to make it a fundamental experience of older adults, especially in aged care where autonomy is often undermined. If we care about their happiness, doing so is arguably a moral imperative.

## Supplementary Material

gnad135_suppl_Supplementary_Material

## Data Availability

The study design for this systematic review was preregistered on the Open Science Framework on May 26, 2022 (https://osf.io/vxkyc/). The R code and data underlying these analyses have been made available is also available via this link. We did not preregister our study with PROSPERO because they are increasingly only accepting meta-analyses that contain clinical dependent variables, which our study did not. Apart from the first and last authors, all other contributors are listed in alphabetical order because all contributions are considered equal.
