# Supplementary material for: A Quantitative Meta-Analysis and Qualitative Meta-Synthesis of Aged Care Residents’ Experiences of Autonomy, Being Controlled, and Optimal Functioning
Source: Gerontologist. 2023 Oct 5;64(5):gnad135. doi: 10.1093/geront/gnad135 (PMC11032117; doi:10.1093/geront/gnad135)
Supplement: gnad135_suppl_Supplementary_Material [file gnad135_suppl_supplementary_material.docx]

**Online Supplementary Material**

**S1.**

*The Search Terms Used in the Systematic Review of the Literature*

**Search Terms**

Titles and abstracts were searched using the following search terms: “self-determination theory” OR “psychological needs” OR “self-determination” OR “autonomy support” or “autonomy-support” OR “psychological control” (separated by the Boolean operator) AND “old* adults” OR “late life” OR “elderly” OR “old* people” OR “aged care” OR “nursing home” OR “elder care” OR “residential care”.

**S2.**

*The Preferred Reporting Items for Systematic Reviews and Meta-Analyses (PRISMA) checklist*

| **Section and Topic** | **Item #** | **Checklist item** | **Done** | **Location where item is reported** |
| --- | --- | --- | --- | --- |
| **TITLE** | | |  |  |
| Title | 1 | Identify the report as a systematic review. | ✓ | p. 3, p. 7 |
| **ABSTRACT** | | |  |  |
| Abstract | 2 | See the PRISMA 2020 for Abstracts checklist. | ✓ | p. 3 |
| **INTRODUCTION** | | |  |  |
| Rationale | 3 | Describe the rationale for the review in the context of existing knowledge. | ✓ | pp. 4-5 |
| Objectives | 4 | Provide an explicit statement of the objective(s) or question(s) the review addresses. | ✓ | p. 5 |
| **METHODS** | | |  |  |
| Eligibility criteria | 5 | Specify the inclusion and exclusion criteria for the review and how studies were grouped for the syntheses. | ✓ | pp. 7-8 |
| Information sources | 6 | Specify all databases, registers, websites, organisations, reference lists and other sources searched or consulted to identify studies. Specify the date when each source was last searched or consulted. | ✓ | p. 8 |
| Search strategy | 7 | Present the full search strategies for all databases, registers and websites, including any filters and limits used. | ✓ | pp. 7-8 and Supplementary Material S1 |
| Selection process | 8 | Specify the methods used to decide whether a study met the inclusion criteria of the review, including how many reviewers screened each record and each report retrieved, whether they worked independently, and if applicable, details of automation tools used in the process. | ✓ | p. 8 |
| Data collection process | 9 | Specify the methods used to collect data from reports, including how many reviewers collected data from each report, whether they worked independently, any processes for obtaining or confirming data from study investigators, and if applicable, details of automation tools used in the process. | ✓ | Supplementary Material S3 and S4 |
| Data items | 10a | List and define all outcomes for which data were sought. Specify whether all results that were compatible with each outcome domain in each study were sought (e.g. for all measures, time points, analyses), and if not, the methods used to decide which results to collect. | ✓ | p. 7 |
|  | 10b | List and define all other variables for which data were sought (e.g. participant and intervention characteristics, funding sources). Describe any assumptions made about any missing or unclear information. | ✓ | Supplementary Material S3 |
| Study risk of bias assessment | 11 | Specify the methods used to assess risk of bias in the included studies, including details of the tool(s) used, how many reviewers assessed each study and whether they worked independently, and if applicable, details of automation tools used in the process. | ✓ | Supplementary Material S3 |
| Effect measures | 12 | Specify for each outcome the effect measure(s) (e.g. risk ratio, mean difference) used in the synthesis or presentation of results. | ✓ | p. 9 |
| Synthesis methods | 13a | Describe the processes used to decide which studies were eligible for each synthesis (e.g. tabulating the study intervention characteristics and comparing against the planned groups for each synthesis (item #5)). | ✓ | p. 8 |
|  | 13b | Describe any methods required to prepare the data for presentation or synthesis, such as handling of missing summary statistics, or data conversions. | ✓ | p. 9 |
|  | 13c | Describe any methods used to tabulate or visually display results of individual studies and syntheses. | ✓ | p. 10 |
|  | 13d | Describe any methods used to synthesize results and provide a rationale for the choice(s). If meta-analysis was performed, describe the model(s), method(s) to identify the presence and extent of statistical heterogeneity, and software package(s) used. | ✓ | pp. 9-10 |
|  | 13e | Describe any methods used to explore possible causes of heterogeneity among study results (e.g. subgroup analysis, meta-regression). | ✓ | p. 9 |
|  | 13f | Describe any sensitivity analyses conducted to assess robustness of the synthesized results. | NA |  |
| Reporting bias assessment | 14 | Describe any methods used to assess risk of bias due to missing results in a synthesis (arising from reporting biases). | NA |  |
| Certainty assessment | 15 | Describe any methods used to assess certainty (or confidence) in the body of evidence for an outcome. | NA |  |
| **RESULTS** | | |  |  |
| Study selection | 16a | Describe the results of the search and selection process, from the number of records identified in the search to the number of studies included in the review, ideally using a flow diagram. | ✓ | Figure 2 |
|  | 16b | Cite studies that might appear to meet the inclusion criteria, but which were excluded, and explain why they were excluded. | NA |  |
| Study characteristics | 17 | Cite each included study and present its characteristics. | ✓ | Supplementary Material S5 |
| Risk of bias in studies | 18 | Present assessments of risk of bias for each included study. | ✓ | Available in the data available online |
| Results of individual studies | 19 | For all outcomes, present, for each study: (a) summary statistics for each group (where appropriate) and (b) an effect estimate and its precision (e.g. confidence/credible interval), ideally using structured tables or plots. | ✓ | Figure 4 and 5 |
| Results of syntheses | 20a | For each synthesis, briefly summarise the characteristics and risk of bias among contributing studies. | ✓ | pp. 11-13 |
|  | 20b | Present results of all statistical syntheses conducted. If meta-analysis was done, present for each the summary estimate and its precision (e.g. confidence/credible interval) and measures of statistical heterogeneity. If comparing groups, describe the direction of the effect. | ✓ | pp. 11-13 |
|  | 20c | Present results of all investigations of possible causes of heterogeneity among study results. | ✓ | pp. 11-13 |
|  | 20d | Present results of all sensitivity analyses conducted to assess the robustness of the synthesized results. | NA |  |
| Reporting biases | 21 | Present assessments of risk of bias due to missing results (arising from reporting biases) for each synthesis assessed. | NA |  |
| Certainty of evidence | 22 | Present assessments of certainty (or confidence) in the body of evidence for each outcome assessed. | NA |  |
| **DISCUSSION** | | |  |  |
| Discussion | 23a | Provide a general interpretation of the results in the context of other evidence. | ✓ | pp. 16-20 |
|  | 23b | Discuss any limitations of the evidence included in the review. | ✓ | p. 20 |
|  | 23c | Discuss any limitations of the review processes used. | ✓ | pp. 20-21 |
|  | 23d | Discuss implications of the results for practice, policy, and future research. | ✓ | p. 21 |
| **OTHER INFORMATION** | | |  |  |
| Registration and protocol | 24a | Provide registration information for the review, including register name and registration number, or state that the review was not registered. | ✓ | p. 7 |
|  | 24b | Indicate where the review protocol can be accessed, or state that a protocol was not prepared. | ✓ | p. 7 |
|  | 24c | Describe and explain any amendments to information provided at registration or in the protocol. | NA |  |
| Support | 25 | Describe sources of financial or non-financial support for the review, and the role of the funders or sponsors in the review. | NA |  |
| Competing interests | 26 | Declare any competing interests of review authors. | NA |  |
| Availability of data, code and other materials | 27 | Report which of the following are publicly available and where they can be found: template data collection forms; data extracted from included studies; data used for all analyses; analytic code; any other materials used in the review. | ✓ | p. 7 |

**S3.**

*Description of the Data Extraction Methods and Moderator Coding Strategy Applied for the Quantitative Meta-Analysis and Qualitative Meta-Synthesis.*

**Data Extraction**

***Quantitative***

Following full-text screening, six of seven co-authors collaborated to extract data from the included quantitative studies. To ensure data quality, after extraction, we randomly selected 20% of the rows in the data extraction table and had a different extractor review the data in each cell. Of the total 12,150 cells in the data extraction table, 2,430 cells were reviewed (20%). Corrections were required in only 0.3% of cases reviewed. Those corrections were made where necessary, prior to analysis.

**Moderator Coding Strategy.** Each effect size extracted was coded as reflecting either a measure of ‘Autonomy’ or a measure of ‘Control’. We used this binary code to calculate one model for each. Then, the effects were coded for their ‘Autonomy Type’ or their ‘Control Type’. Autonomy Type comprised five levels: autonomy support, autonomous motivation, identified motivation, intrinsic motivation, and basic psychological need satisfaction. Control Type also comprised five levels: controlled motivation, amotivation, external motivation, introjected motivation, and basic psychological need frustration. The effects were then coded for ‘Outcome Type’ according to whether they were assessing the link between Autonomy or Control and an index of (a) psychological wellbeing (e.g., life satisfaction, positive affect), (b) physical wellbeing (e.g., subjective physical health, pain, energy levels, physical activity), or (c) healthy reliance (e.g., adaptation to nursing home life, “I have good contact with the people who work at the residence”, Castonguay & Ferron, 1999). Psychological wellbeing effects were coded as either wellbeing (e.g., satisfaction with life) or ill-being (e.g., depression). All ill-being effects were then reversed to be pooled with wellbeing and healthy reliance effects and referred to, en masse, as wellness outcomes.

In terms of demographic variables, we extracted data pertaining to ‘Country’ of which there were six: Belgium, Canada, China, France, the Netherlands, and Taiwan. There was one study from Australia also, though it was a longitudinal study, and there were too few longitudinal studies to reliably to meta-analyze the mean standardized difference. We discuss the longitudinal studies narratively. We also extracted the proportion of females and the total sample size. These variables were used to calculate the proportion of females in the samples, so that we could use it as a continuous moderating variable. Finally, we extracted average age and changed it to a variable indexing the years since age 65, to assess age as a moderator.

***Qualitative***

Following full-text screening, three co-authors collaborated on the qualitative data extraction strategy using Nvivo. We searched the full texts for every explicit instance of our keywords (i.e., autonomy/self-determination—(*n* = 859, *n* = 255), control—(*n* = 168), and proxies for healthy reliance), using the word frequency function. From the text searches we extracted all definitions/descriptions of autonomy/self-determination (*n* =, *n* =, control (*n* =), and proxies for healthy reliance (*n* = ).

Because ‘healthy reliance’ and/or ‘healthy adaptation’ to nursing home life are specific terms, we did not expect to find them explicitly in the texts and instead used frequently appearing synonyms to direct us to the relevant qualitative data. We reviewed the appearances of ‘support’ (*n* = 289), ‘socially’ (*n* = 262), ‘relationships’ (*n* = 221), ‘helps’ (*n* = 178), ‘relatedness’ (*n* = 172), ‘community’ (*n* = 153), and ‘loneliness’ (*n* = 131) to detect resident and researcher definitions and descriptions of healthy reliance. The results sections from all qualitative papers were also read in full to ensure all relevant data had been captured.

**S4.**

*Description of the Methods Used to Evaluate Study Quality for the Quantitative Meta-Analysis and Qualitative Meta-Synthesis.*

**Study Quality**

***Quantitative***

**Risk of Bias.** Consistent with recent SDT meta-analyses (Bradshaw et al., 2022), we rated each report on four binary indicators of study quality: (a) was the participant eligibility criteria clear and specific?, (b) was the sample representative of the population?, (c) did the study use a valid measure of intrinsic and extrinsic aspirations?, and, (d) did the study use a valid measure of wellbeing and/or ill-being? Risk of bias scores of 1 indicated high risk of bias, 2-3 indicated moderate risk of bias, and 4 indicated low risk of bias. We then included the three-level risk of bias variable as a moderator in all our analyses.

**Publication Bias.** The jury is still out regarding the most effective means of evaluating publication bias in multi-level meta-analyses, with the plethora of available tests each offering pros and cons (Rodgers & Pustejovsky, 2021). Carter et al. (2019) recommends combining multiple methods to shine sufficient light on possible sources of bias. Accordingly we used Egger’s multi-level regression test (Fernández-Castilla et al., 2021; Rodgers & Pustejovsky, 2021) combined with visual inspection of aggregated funnel plots to assess publication bias. Egger’s multi-level regression tests the link between the effect sizes and standard errors. If meta-analytic effects are correlated with their standard errors it can be evidence of publication bias and a sign that some statistically nonsignificant effects have not been published. Funnel plot asymmetries can further inform the presence of such bias.

***Qualitative***

We evaluated the quality of the qualitative reports in our review using the 10-item Critical Appraisal Skills Program (CASP, 2013). The measure assesses the clarity of the study’s aim/s (a), appropriateness of the method (b), research design (c), and participant recruitment (d), effectiveness (e) and rigor (f) of the data use, acknowledgement of potential bias (g) and ethical considerations (h), and whether the results are clearly communicated (i) and valuable (j). As per previous studies, three authors collaborated to rate each of these indices as a zero if they were not met, a one if they addressed but weakly, or a two if they were satisfactorily addressed (Boeije et al., 2011; Lachal et al., 2017). Study quality varied across the studies included in this review, though none were of poor enough quality to compromise the utility of the results, nor to warrant exclusion. Therefore, no studies were excluded because of the quality assessments.

**S5.**

*A Summary of the Studies Included in the Meta-Analyses, and Their Effects, Divided According to the Autonomy and Control Models*

| Author/s | Year | Title | Study | *n* | Female (%) | *k* | *r* |
| --- | --- | --- | --- | --- | --- | --- | --- |
| **Autonomy & Wellness Outcomes** | | |  |  |  |  |  |
| Altintas et al. | 2017 | Adaptation to nursing home: The role of leisure activities in light of motivation and relatedness | 1 | 112 | 82.14 | 6 | 0.34 – 0.58 |
| Altintas & Guerrien | 2009 | Orientation motivationnelle et symptomatologie dépressive chez la personne âgée [Motivational orientation and depressive symptoms in the elderly] | 1 | 125 | 24.80 | 2 | -0.27 –  -0.42 |
| Altintas et al. | 2018 | Leisure activities and motivational profiles in adaptation to nursing homes | 1 | 113 | 82.30 | 8 | 0.16 – 0.57 |
| Altintas et al. | 2010 | Adaptation des aînés à la résidence pour personnes âgées: Activité de loisirs et autodétermination [Adaptation of seniors in nursing homes: Activity, leisure, and self-determination] | 1 | 77 | 80.52 | 6 | 0.12 – 0.48 |
| Buckinx et al. | 2020 | The effects of GAMotion (a giant exercising board game) on physical capacity, motivation, and quality of life among nursing home residents: A pilot interventional study | 1 | 21 | 47.62 | 3 | SMD |
| Chang | 2018 | Is social support always related to stress reduction in nursing home residents?: A study in leisure contexts | 1 | 139 | 63.31 | 1 | -0.26 |
| Custers et al. | 2014 | Need fulfillment, need importance, and depressive symptoms of residents over the first eight months of living in a nursing home | 1 | 73 | 58.90 | 6 | -0.55 – 0.52 |
| Davison et al. | 2021 | The effectiveness of the Program to Enhance Adjustment to Residential Living (PEARL) in reducing depression in newly admitted nursing home residents | 1 | 216 | 64.81 | 3 | SMD |
| Davison et al. | 2022 | Program to Enhance Adjustment to Residential Living (PEARL): Effect on adjustment, anxiety, quality of life, and stress | 1 | 216 | 64.81 | 12 | SMD |
| Ferrand et al. | 2019 | Need satisfaction and frustration in older people living in French nursing homes | 1 | 134 | 64.93 | 6 | -0.26 – 0.42 |
| Fortin et al. | 2001 | Suicidal ideation and self-determination in institutionalized elderly | 1 | 22 | NA | 2 | -0.46 –  -0.44 |
| Kloos et al. | 2019 | Longitudinal associations of autonomy, relatedness, and competence with the well-being of nursing home residents | 1 | 128 | 72.66 | 6 | -0.55 - 0.43 |
| O'Connor & Vallerand | 1994a | Motivation, self-determination, and person-environment fit as predictors of psychological adjustment among nursing home residents | 1 | 129 | 86.05 | 4 | 0.04 – 0.57 |
| O'Connor & Vallerand | 1994b | The relative effects of actual and experienced autonomy on motivation in nursing home residents | 1 | 129 | 86.05 | 3 | 0.04 – 0.07 |
| Paque et al. | 2017 | Autonomy and social functioning of recently admitted nursing home residents | 1 | 391 | 64.45 | 7 | -0.26 – 0.28 |
| Souesme et al. | 2016 | Perceived autonomy support, psychological needs satisfaction, depressive symptoms and apathy in French hospitalized older people | 1 | 100 | 69 | 8 | -0.59 –  -0.32 |
| Tang et al. | 2021 | Effect of “freedom of choice” on task performance and well-Being during leisure activity: An intercultural study among older adults in China and France | 1 | 67 | 52.22 | 9 | -0.36 - 0.41 |
| Tang et al. | 2021 | Effect of “freedom of choice” on task performance and well-Being during leisure activity: An intercultural study among older adults in China and France | 1 | 90 | 86.67 | 9 | -0.16 - 0.53 |
| Vanroy et al. | 2019 | Can a framed intervention motivate older adults in assisted living facilities to exercise? | 1 | 99 | 67.67 | 2 | 0.22 – 0.28 |
| **Control & Wellness Outcomes** | | |  |  |  |  |  |
| Altintas et al. | 2017 | Adaptation to nursing home: The role of leisure activities in light of motivation and relatedness | 1 | 112 | 82.14 | 4 | -0.39 – 0.40 |
| Altintas & Guerrien | 2009 | Orientation motivationnelle et symptomatologie dépressive chez la personne âgée [Motivational orientation and depressive symptoms in the elderly] | 1 | 125 | 24.80 | 2 | 0.41 – 0.50 |
| Altintas et al. | 2018 | Leisure activities and motivational profiles in adaptation to nursing homes | 1 | 113 | 82.30 | 8 | -0.36 – 0.13 |
| Altintas et al. | 2010 | Adaptation des aînés à la résidence pour personnes âgées: Activité de loisirs et autodétermination [Adaptation of seniors in nursing homes: Activity, leisure, and self-determination] | 1 | 77 | 80.52 | 9 | -0.40 – 0.25 |
| Ferrand & Martinent | 2021 | Need frustration and depressive symptoms in French older people: Using a self-determination approach | 1 | 116 | 46.55 | 3 | 0.21 – 0.33 |
| Ferrand et al. | 2019 | Need satisfaction and frustration in older people living in French nursing homes | 1 | 134 | 64.93 | 6 | -0.23 – 0.38 |
| O'Connor & Vallerand | 1994a | Motivation, self-determination, and person-environment fit as predictors of psychological adjustment among nursing home residents | 1 | 129 | 86.05 | 4 | -0.53 – 0.01 |
| Vanroy et al. | 2019 | Can a framed intervention motivate older adults in assisted living facilities to exercise? | 1 | 99 | 67.67 | 2 | 0.08 – 0.13 |

*Note*. *n* = number of participants; *k* = number of effects; correlations with ill-being outcomes (e.g., for depression etc.) were reversed in the analysis such that all outcomes served as positive indicators of wellness; SMD = standardized mean differences were calculated based on treatment and control groups’ means and standard deviations, for more specificity regarding effect size reversal and the calculation of SMD, please see the full data extraction table available via the OSF link in the manuscript.

**S6.**

*A Summary of the Studies Included in the Qualitative Meta-Synthesis*

| Author/s | Year | Title | Study | *n* | Female (%) | *k* | Variable/s |
| --- | --- | --- | --- | --- | --- | --- | --- |
| Andersson et al. | 2007 | Daily life after moving into a care home – experiences from older people, relatives and contact persons | 1 | 13 | 45.16 | 8 | Autonomy, Control |
| Bollig et al. | 2016 | Nothing to complain about? Residents’ and relatives’ views on a “good life” and ethical challenges in nursing homes | 1 | 25 | 64 | 13 | Autonomy, Control, Autonomous Reliance |
| Hellström & Sarvimäki | 2007 | Experiences of self-determination by older persons living in sheltered housing | 1 | 11 | 54.54 | 12 | Autonomy, Control, Autonomous Reliance |
| Kaelen et al. | 2021 | How to bring residents' psychosocial well-being to the heart of the fight against Covid-19 in Belgian nursing homes—A qualitative study | 1 | 56 | 62.5 | 2 | Control |
| MacLeod | 2018 | A phenomenological exploration of autonomy and related psychological needs among the residents of a memory care unit | 1 | 5 | 80 | 10 | Autonomy, Autonomous Reliance |
| Nakrem et al. | 2011 | Residents’ experiences of interpersonal factors in nursing home care: A qualitative study | 1 | 15 | 60 | 9 | Autonomy, Control, Autonomous Reliance |
| Nusbaum | 2011 | How the elder co-housing model of living affects residents' experience of autonomy: A self-determination theory perspective | 1 | 10 | 60 | 2 | Autonomy |
| Paque et al. | 2018 | Living in a nursing home: A phenomenological study exploring residents’ loneliness and other feelings | 1 | 11 | 63.64 | 5 | Autonomy, Control |
| Schenell et al. | 2020 | Struggling for a dignified life: The meaning of self-determination in palliative phase in residential care | 1 | 20 | 60 | 18 | Autonomy, Control, Autonomous Reliance |
| Schenk et al. | 2013 | Quality of life in nursing homes: Results of a qualitative resident survey | 1 | 42 | 78.57 | 3 | Autonomy, Autonomous Reliance |
| Schmidt et al. | 2018 | Needs of people with advanced dementia in their final phase of life: A multi-perspective qualitative study in nursing homes | 1 | 30 | 76.67 | 2 | Autonomy |

*Note*. *n* = number of participants, *k* = number of qualitative data items

**S7.**

*A Funnel Plot of the Effects Linking Residents’ Experiences of Autonomy with Their Wellness Outcomes*


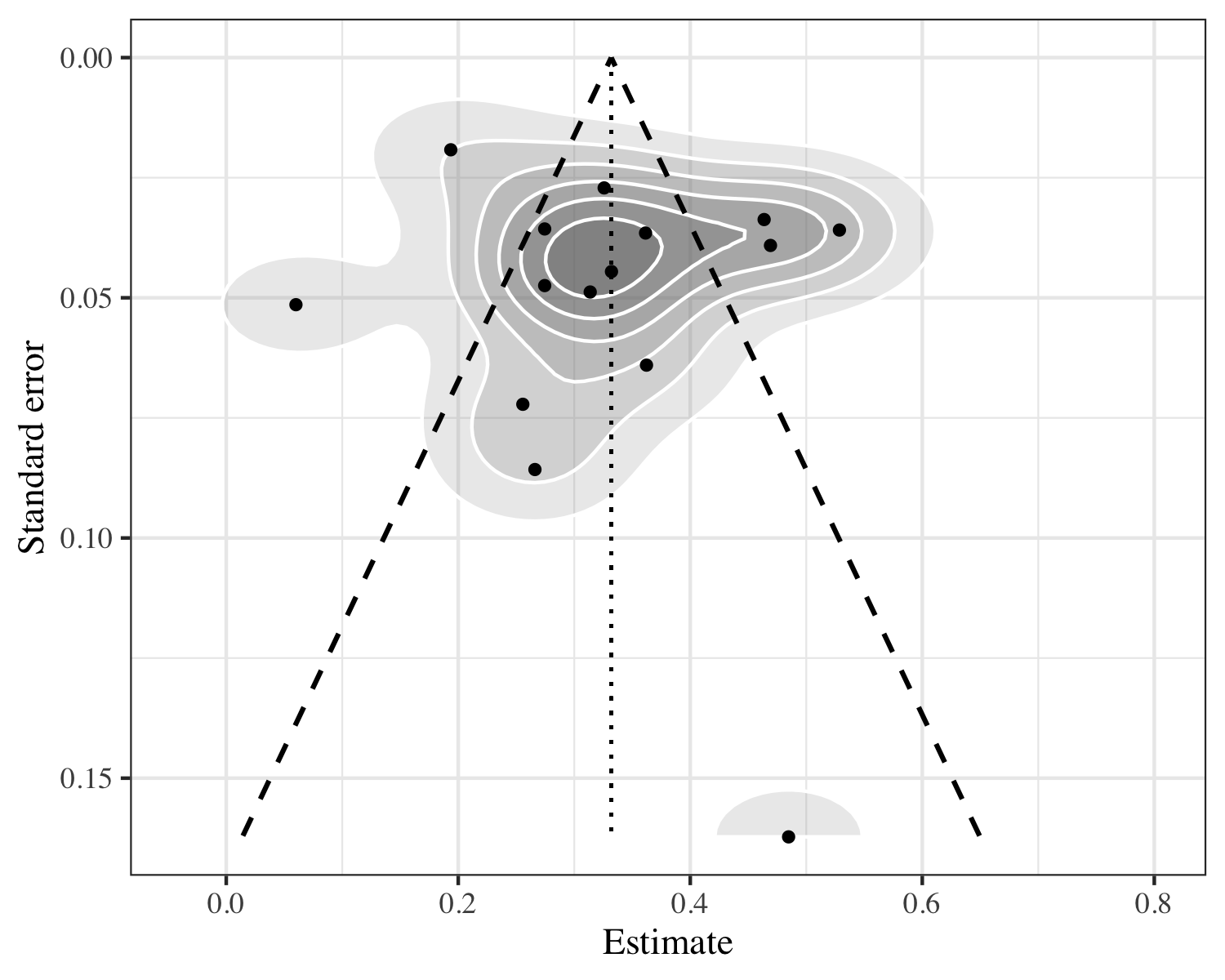


**S8.**

*A Funnel Plot of the Effects Linking Residents’ Experiences of Control with Their Wellness Outcomes*


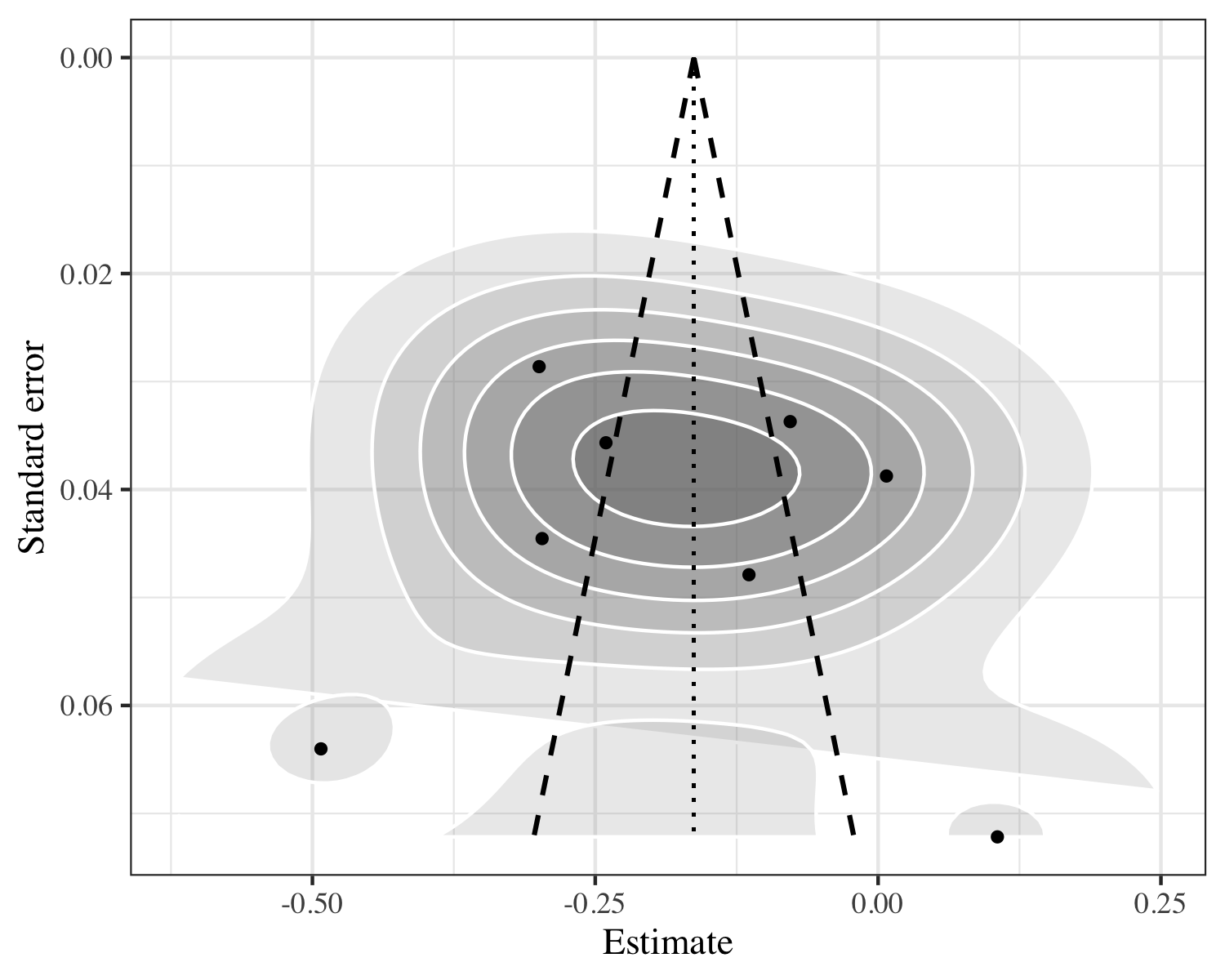


**Supplementary References**

Boeije, H. R., van Wesel, F., & Alisic, E. (2011). Making a difference: Towards a method for weighing the evidence in a qualitative synthesis. *Journal of Evaluation in Clinical Practice, 17*(4), 657-663. <https://doi.org/https://doi.org/10.1111/j.1365-2753.2011.01674.x>

Bradshaw, E. L., Conigrave, J. H., Steward, B. A., Ferber, K. A., Parker, P. D., & Ryan, R. M. (2022). A meta-analysis of the dark side of the American dream: Evidence for the universal wellness costs of prioritizing extrinsic over intrinsic goals. *Journal of Personality and Social Psychology*. <https://doi.org/https://doi.org/10.1037/pspp0000431>

Castonguay, G., & Ferron, S. (1999). Elaboration et validation d'un instrument evaluant le degre d'adaptation de la personne agee a sa residence [Development and validation of an instrument evaluating the degree of adaptation of the elderly person to his residence]. *Canadian Journal on Aging, 18*(3), 363-375. <https://doi.org/10.1017/S0714980800009922>

Critical Appraisal Skills Program. (2013). *Qualitative research checklist*. Retrieved from: <http://docs.wixstatic.com/ugd/dded87_25658615020e427da194a325e7773d42.pdf>

Lachal, J., Revah-Levy, A., Orri, M., & Moro, M. R. (2017). Metasynthesis: An original

method to synthesize qualitative literature in psychiatry. *Frontiers in Psychiatry*, *8*, 269. [https://doi.org/https://doi.org/10.3389/fpsyt.2017.00269](https://doi.org/https:/doi.org/10.3389/fpsyt.2017.00269)

Rodgers, M. A., & Pustejovsky, J. E. (2021). Evaluating meta-analytic methods to detect

selective reporting in the presence of dependent effect sizes. *Psychological Methods, 26*(2), 141-160. <https://doi.org/10.1037/met0000300>
